# Alpha Power Modulates Perception Independently of Endogenous Factors

**DOI:** 10.3389/fnins.2018.00279

**Published:** 2018-04-25

**Authors:** Sasskia Brüers, Rufin VanRullen

**Affiliations:** ^1^UMR 5549, Faculté de Médecine Purpan, Centre National de la Recherche Scientifique, Toulouse, France; ^2^Centre de Recherche Cerveau et Cognition, Université de Toulouse, Université Paul Sabatier, Toulouse, France

**Keywords:** alpha oscillations, power, EEG, visual perception, IRF

## Abstract

Oscillations are ubiquitous in the brain. Alpha oscillations in particular have been proposed to play an important role in sensory perception. Past studies have shown that the power of ongoing EEG oscillations in the alpha band is negatively correlated with visual outcome. Moreover, it also co-varies with other endogenous factors such as attention, vigilance, or alertness. In turn, these endogenous factors influence visual perception. Therefore, it remains unclear how much of the relation between alpha and perception is indirectly mediated by such endogenous factors, and how much reflects a direct causal influence of alpha rhythms on sensory neural processing. We propose to disentangle the direct from the indirect causal routes by introducing modulations of alpha power, independently of any fluctuations in endogenous factors. To this end, we use white-noise sequences to constrain the brain activity of 20 participants. The cross-correlation between the white-noise sequences and the concurrently recorded EEG reveals the impulse response function (IRF), a model of the systematic relationship between stimulation and brain response. These IRFs are then used to reconstruct rather than record the brain activity linked with new random sequences (by convolution). Interestingly, this reconstructed EEG only contains information about oscillations directly linked to the white-noise stimulation; fluctuations in attention and other endogenous factors may still modulate brain alpha rhythms during the task, but our reconstructed EEG is immune to these factors. We found that the detection of near-perceptual threshold targets embedded within these new white-noise sequences depended on the power of the ~10 Hz reconstructed EEG over parieto-occipital channels. Around the time of presentation, higher power led to poorer performance. Thus, fluctuations in alpha power, induced here by random luminance sequences, can directly influence perception: the relation between alpha power and perception is not a mere consequence of fluctuations in endogenous factors.

## Introduction

When recording the electro-encephalography (EEG) in humans, one of the most prominent rhythm is the ~ 10 Hz oscillations over the occipito-parietal cortex. Most current theories agree that alpha oscillations play an active inhibitory role in shaping our visual experience (Klimesch et al., 2007; Jensen and Mazaheri, 2010; Foxe and Snyder, 2011; Mathewson et al., 2011; VanRullen, 2016).

Evidence in line with these theories has come from studies linking the instantaneous power of alpha oscillations over the occipito-parietal channels to visual outcome. For example, Ergenoglu et al. (2004) showed that stronger power in the ~10 Hz ongoing EEG oscillation before target presentation led to poorer detection performance (Ergenoglu et al., 2004). This correlation between trial-by-trial variability in alpha amplitude and visual performance has since then received support from various other experimental paradigms (van Dijk et al., 2008; Busch et al., 2009; Wyart and Tallon-Baudry, 2009; Mathewson et al., 2011).

At the neuronal level, these changes in alpha power reflect modulations of excitability. In fact, the instantaneous level of alpha oscillatory power is a good index of the excitability of the cortex measured by single pulse transcranial magnetic stimulation (TMS) between (Romei et al., 2008a) as well as within subjects: participants were more likely to report a “phosphene” (illusory percept) when the ongoing EEG alpha power was (relatively) lower (Romei et al., 2008a, replicated by Dugué et al., 2011 and Samaha et al., 2017). The functional role of these spontaneous fluctuations in excitability of the cortex is still unresolved: they could be used for the detection of unpredictable events in the visual field through a spatial scanning mechanism (Romei et al., 2008a).

In fact, the power of alpha oscillations also co-varies with endogenous factors (such as attention), as uncovered using the classical Posner paradigm: a central cue (usually an arrow) is used to induce the deployment of covert attention (i.e., without eye movement) toward a visual hemi-field in preparation for the upcoming peripheral target (Posner, 1980). Using this paradigm, studies have shown that the instantaneous strength of the EEG alpha power follows the deployment of spatial attention resulting in two complementary effects with regards to the location of the target: a decreased alpha power over the contra-lateral sensors (Sauseng et al., 2005; Yamagishi et al., 2005; Thut et al., 2006; van Diepen et al., 2015) and/or an increased power over the ipsilateral sensors (Worden et al., 2000; Kelly et al., 2006; Rihs et al., 2007; Cosmelli et al., 2011). Since this power lateralization is retinotopic (Kelly et al., 2006) the locus of spatial attention can be successfully decoded from the topography of the power of alpha oscillations (Samaha et al., 2016).

Importantly, the Posner paradigm has also been used to uncover the effects of endogenous factors on behavior. The attended targets (i.e., presented in the cued hemi-field) are detected faster (Posner, 1980), and more accurately (Posner et al., 1980) than uncued targets. These effects have been extensively reviewed (e.g., Carrasco, 2011; Petersen and Posner, 2012) and replicated: attention acts as a selective tool to narrow the amount of information and optimize the use of our limited brain resources (Carrasco, 2014).

**Figure 1:**
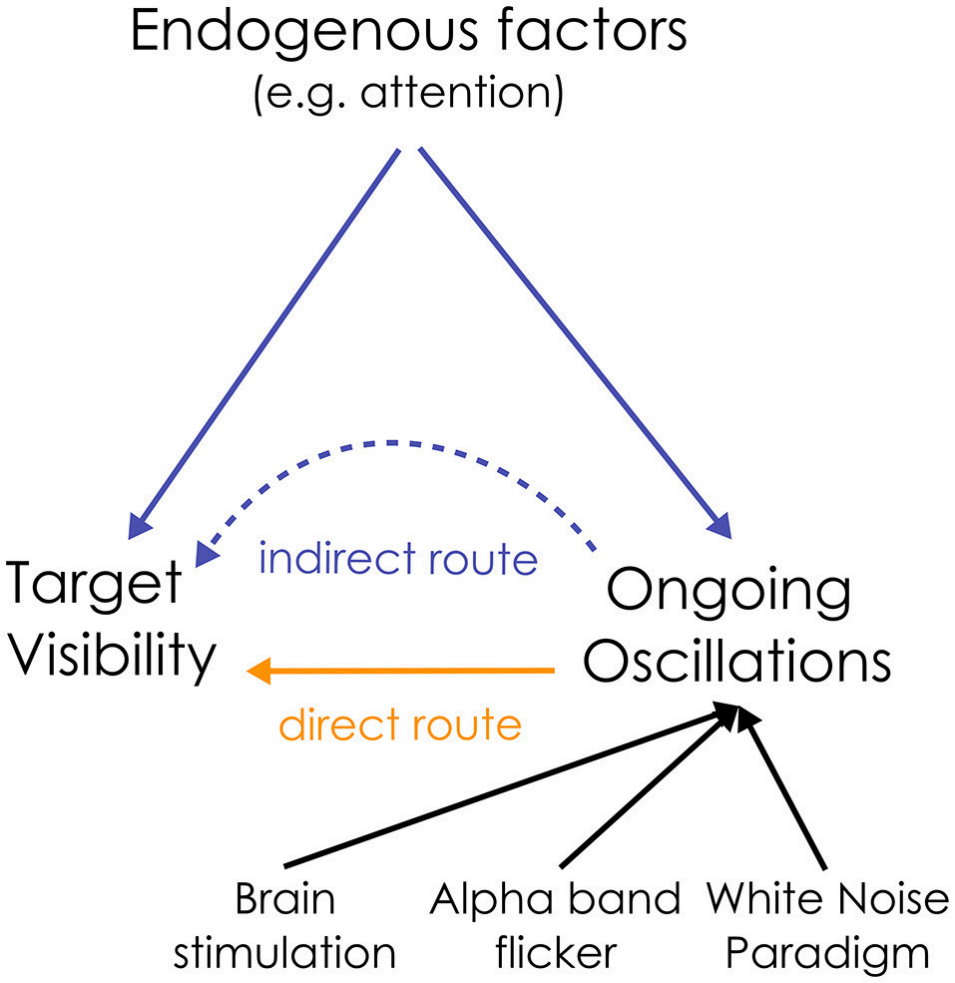
Dual route between the ongoing oscillation power and visual perception. The relationship between alpha oscillations and visual perception could be a direct causal one, or could be mediated by an indirect influence of endogenous factors on both ongoing oscillations and target detection. To disentangle the relative contribution of both routes, the state of the ongoing oscillations can be directly manipulated, e.g., by rhythmic brain or visual stimulation (flicker), to test their causal influence on perception. Here, we propose to use the “White Noise Paradigm”.

To summarize, both visual detection and the power of alpha oscillations are directly influenced by attention: this creates an indirect link between ongoing oscillations and perception (see Figure 1). In this “indirect” route, oscillations have no causal influence on perception. Rather, any measured correlation between oscillations and perception can be attributed to a common driving influence of endogenous factors. How can we disentangle the relative contribution of these two (direct/indirect) causal routes on sensory neural processing?

It is possible to do so by directly modulating the state of the ongoing oscillations, independently of any fluctuations in endogenous factors. By synchronizing the ongoing oscillations with an external driving rhythm, the functional relevance of oscillations can be tested by probing perception as a function of this entrainment (Thut et al., 2011a). Two methods have previously been used to entrain the oscillations: either through transcranial stimulation techniques or through the stimulation of sensory pathways (Thut et al., 2011a), and both methods have been shown to successfully entrain the oscillations for several cycles after the stimulation stopped (Thut et al., 2011b; Halbleib et al., 2012; Helfrich et al., 2014; Spaak et al., 2014).

Crucially, these entrained oscillations have been found to causally influence visual target detection and discrimination. Using rhythmic TMS, Romei et al. (2010) applied a train of pulses over the occipital and parietal cortex in the theta, alpha or the beta band. They found a frequency specific modulation of performance by the entrained alpha rhythm (compared to two frequency bands): the detection of contralateral (near-perceptual threshold) visual targets was decreased concurrently with increased alpha power by the rTMS (Romei et al., 2010). Similarly for tACS, Kanai and colleagues found that stimulation at alpha frequency using tACS was the most effective in inducing an illusory perception (i.e., phosphenes) in the dark (Kanai et al., 2008). In addition, Helfrich et al. (2014) showed that tACS successfully entrained the alpha oscillations over the parieto-occipital cortex. These entrained oscillations successfully modulated behavior (Helfrich et al., 2014).

Using four flashes of light to entrain the oscillations, de Graaf et al. (2013) showed that entrainment of the alpha oscillations (compared with other frequencies) resulted in a specific impairment of the usual cueing benefit (de Graaf et al., 2013). In a separate task, they also found that the discrimination of a target presented after the entraining rhythm was modulated during 3 cycles of the alpha rhythm (de Graaf et al., 2013). This effect was also replicated by Spaak et al. (2014) who showed, in addition, that this rhythmic modulation of performance was supported by a neural entrainment of alpha oscillations.

Thus, these rhythmic transcranial or flickering stimulations have been successfully used to study the direct causal influence of ongoing oscillations on sensory processing. Nevertheless, these methods require an *a priori* hypothesis about the stimulation frequency (Dugué and VanRullen, 2017): the above mentioned studies used 10 Hz (Kanai et al., 2008; Romei et al., 2010; Spaak et al., 2014) and 10.6 HZ (de Graaf et al., 2013) respectively. There is, however, a difficulty in choosing the exact stimulation frequency: each subject has their own individual alpha peak frequency, which varies as a function of the task demands (Haegens et al., 2014; Mierau et al., 2017). Ideally, the stimulation would be tailored to the individual subject's alpha peak frequency to create a 1:1 frequency locking, a condition “ideal” for entrainment (Thut et al., 2011a).

In this study, we use an alternative method which overcomes this limitation of choosing a specific frequency of stimulation. By means of the White Noise (WN) Paradigm (Brüers and VanRullen, 2017), the same stimuli can be used to constrain the state of background oscillations in a subject specific manner. White-noise (random luminance) sequences are used as the driving visual stimulus. These have been shown to create perceptual echoes in the brain (VanRullen and Macdonald, 2012), whose individual peak frequency is highly correlated with the individual alpha peak frequency (VanRullen and Macdonald, 2012). Therefore these WN sequences allow us to modulate the alpha power independently of any fluctuations in endogenous factors. Crucially, they can be used to model this modulated alpha activity, without having to record the EEG. We used them here to investigate whether changes in the state of alpha oscillations can influence target visibility directly, regardless of the attentional state.

## Method

In this study, we re-analyzed data from a previously published dataset (Brüers and VanRullen, 2017). For the convenience of the reader, the necessary information is described again here.

### Participants

Twenty-one volunteers were included in the experiment. However, technical issues during EEG acquisition prevented us from analyzing the data from one participant. Consequently, the data from 20 participants (aged 23–39 years old with a mean age of 28, 10 men, 5 left handed) was analyzed. All subjects reported normal or corrected to normal vision and no history of epileptic seizures or photosensitivity. In accordance with the Declaration of Helsinki, all subjects gave written informed consent before starting the experiment. This study was carried out in accordance with the guidelines for research at the “Centre de Recherche Cerveau et Cognition” and the protocol was approved by the committee “Comité de protection des Personnes Sud Méditerranée 1” (ethics approval number N° 2016-A01937-44).

### Stimuli and protocol

**Figure 2:**
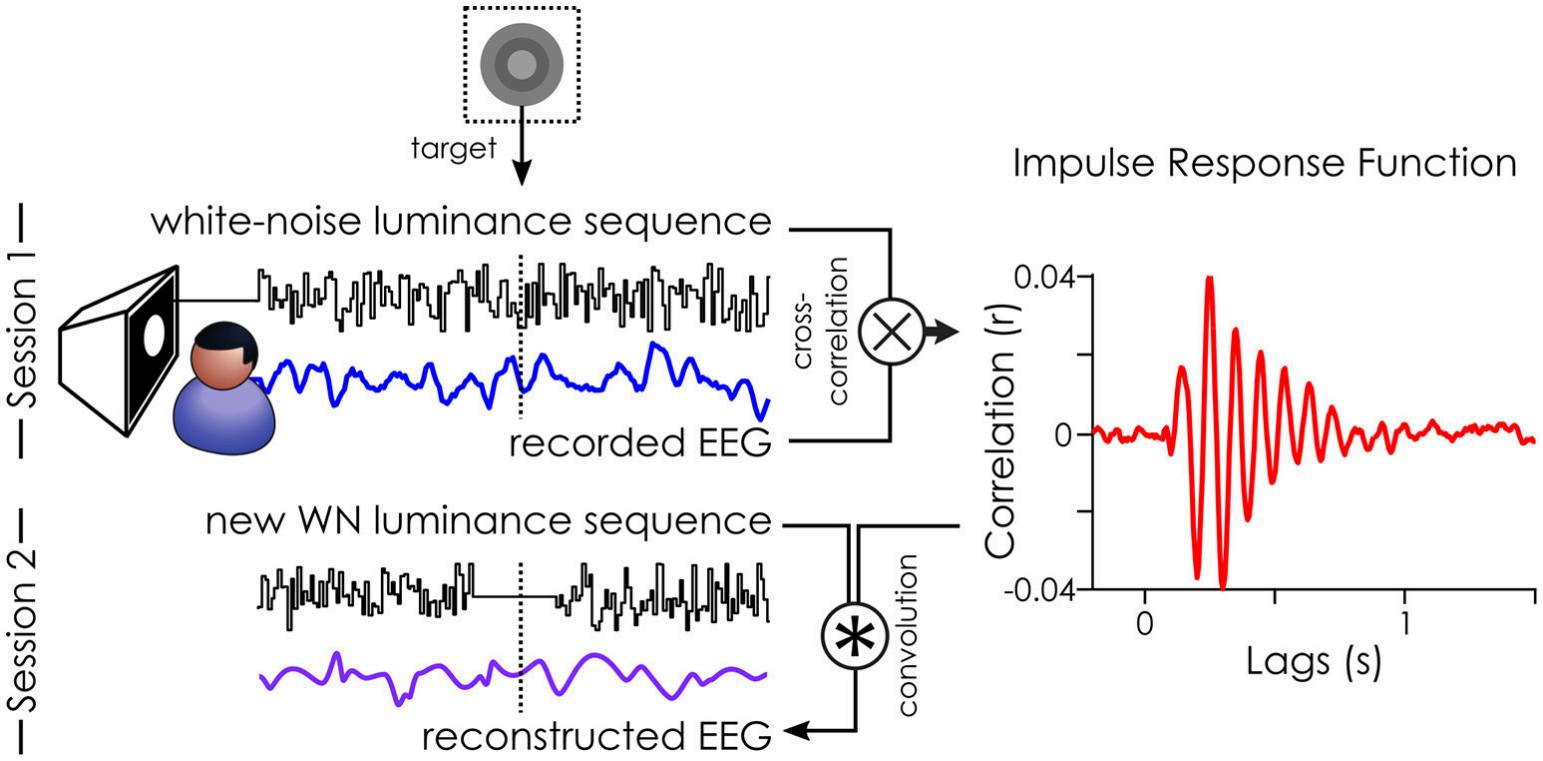
Illustration of the White-Noise Paradigm. The impulse response function (IRF) to white-noise sequences can be extracted by cross-correlating the stimuli sequence with the recorded EEG (done in session 1). Here, an example IRF from one subject on the parieto-central channel. This IRF can, in turn, be used to reconstruct the brain activity (reconstructed EEG) to any new white-noise sequence by convolution (done for session 2). Figure originally published in *eNeuro* in Brüers and VanRullen (2017).

The experiment was composed of two sessions of 8 experimental blocks (of 48 trials), lasting about 1 h each (depending on the duration of the self-administered rests). The stimuli were 6.25 s long random luminance (white-noise) sequences presented on a cathode ray monitor positioned 57 cm from the subject and a resolution of 640 × 480 pixels and refresh rate of 160 Hz. The white-noise sequences had a flat power spectrum up to 80 Hz (on average). The stimuli were created using custom script in MATLAB and presented using the Psychophysics Toolbox (Brainard, 1997). The sequences were presented on a black background in an overhead peripheral disk with a diameter of 7°, whose center was at 7° of eccentricity from fixation. Participants initiated the beginning of each trial and block by a button press. The task was to maintain covert attention to the WN sequences and report the presence of targets (from 2 to 4 targets per trial) embedded within them by pressing a button. The targets (a lighter circle surrounded by a darker ring) were presented for 1 frame only on a medium gray background disk (see Figure 2). Using a staircase procedure, we manipulated the visibility of these targets by changing the contrast between the outer (darker ring) and inner (lighter circle) parts to reach the contrast at which participants perceived about 50% of the first 100 targets presented (i.e., about 30 trials), keeping the resulting contrast constant for the remainder of the session. The perceptual threshold was computed for each session independently using the *quest* function (Watson and Pelli, 1983). Both parts of the target were updated symmetrically so as to keep a “medium gray” mean luminance level for the target frames, identical to the mean “medium gray” level of the white-noise sequence. During the second session, we removed the luminance fluctuations around the presentation of the target, as piloting showed a strong influence of these raw luminance values on perception: higher luminance systematically led to poorer target detection (Brüers and VanRullen, 2017). Thus, 156.25 ms long fluctuations-free periods were created around each target by setting 14 frames before and 11 frames around the target to the same target background “medium gray” value. A control experiment (on 6 independent subjects) revealed that these fluctuation-free periods could not be detected by the subjects (Brüers and VanRullen, 2017).

### Recording and pre-processing EEG data

We recorded the brain activity to WN sequences using a 64 channels active BioSemi electro-encephalography (EEG) system (1,024 Hz digitizing rate, 3 ocular electrodes) in session 1. The following pre-processing steps were applied to all subjects using the EEGlab toolbox (Delorme and Makeig, 2004) in Matlab. Once the noisy channels had been rejected and interpolated (if necessary), the data was down-sampled to 160 Hz (offline) to match presentation rate of stimuli and thus facilitate the cross-correlation of the two signals. A notch filter was then applied (between 47 and 53 Hz) to remove power line artifacts. An average-referencing scheme was applied and slow drifts were removed from the data by applying a high-pass filter (>1 Hz). Data epochs (384) were created around each white-noise sequence (from −0.25 to 6.5 s) and the baseline activity was removed (i.e., mean activity from −0.25 s to 0 before trial onset). Finally, the data was screened manually for eye movements, blinks and muscular artifacts and whole epochs were rejected as needed: on average 20/384 trials were rejected per subject.

### Extracting IRF and reconstructing the EEG

The pre-processed and z-scored EEG data was cross-correlated with the z-scored white-noise sequences (see Figure 2) to extract the impulse response functions (IRF, also called VESPA by Lalor et al., 2006; or “perceptual echoes” by VanRullen and Macdonald, 2012). For each subject, this created one IRF for each of 64 EEG channels.

In session 2, we created a new set of white-noise sequences which we presented to all subjects in a randomized order. This allowed us to directly compare how the same targets (across subjects) would be perceived. Here, we only collected the behavioral responses to these new white-noise sequences. Instead of recording the brain activity (“recorded EEG”), we estimated it (“reconstructed EEG”) by doing a convolution between the IRFs (as a model of brain response) and the white-noise sequences presented in session 2. The mean target luminance (“medium gray”) was included in the reconstruction. As such, it was no different from the surrounding values in the sequence, and thus, no ERP was evoked by the target in the reconstructed EEG (see Figure 5 in Brüers and VanRullen, 2017). We created 1.6 s long epochs of reconstructed EEG around each target ([−800 ms – +794 ms]). Any epoch where the stimulation was faulty (i.e., target was presented for more than one frame) or when the staircase had not converged (i.e., first 100 targets) was removed from analysis for all subjects, yielding 821 acceptable trials. The oscillatory characteristics (power and phase) of the reconstructed EEG were extracted using a time frequency transform (using 46 wavelets varying from 3 to 75 Hz in log-spaced frequency steps with 2–8 cycles).

### Computing the power difference between conditions

**Figure 3:**
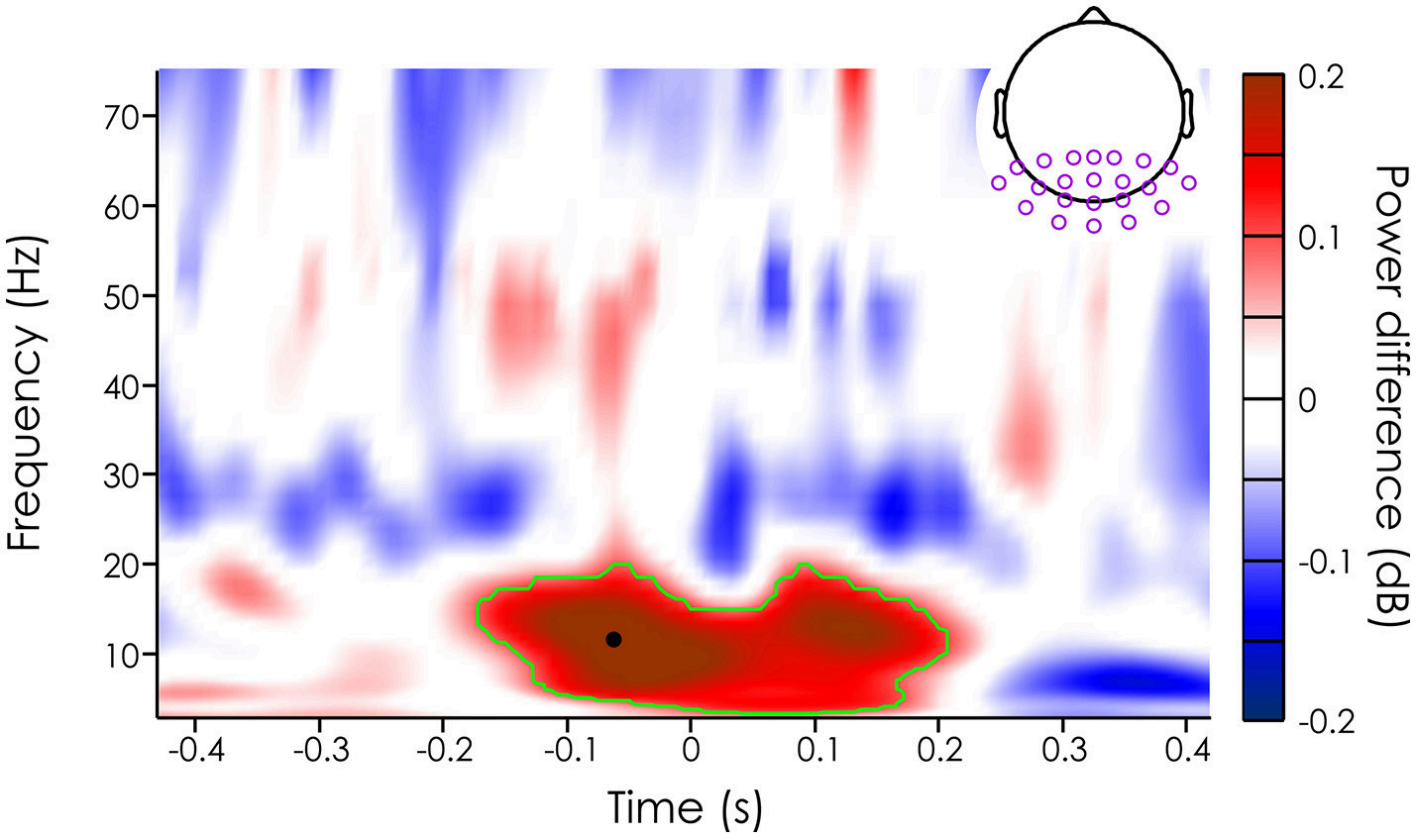
Mean power difference (in dB [unseen-seen]) averaged across subjects (*N* = 20) and channels in the ROI (purple dots on the topography). The green outline represents the significant cluster after cluster correction (see Methods section). The black dot represents the peak power difference (11.68 Hz and −62.5 ms).

To test whether the power of the reconstructed EEG had an impact on behavior, we evaluated the difference between the power of hits and missed target epochs. We restricted our analysis to a region of interest (ROI) based on the localization of previous effects of power reported in previous studies. This ROI was composed of 22 channels in the occipital-parietal region including all parietal, parieto-occipital and occipital channels (purple dots on Figure 3). A decibel difference (dB) was computed for each subject at each channel, frequency and time point as the log transformed ratio between the mean power of undetected target trials (misses) and the mean power of detected target trials (hits). We compared this “real” power difference to a “surrogate” distribution. We created 1,000 surrogates per subject by systematically switching labels between conditions (hits and misses), and recomputing the power difference between these arbitrary trial groups for each channel, time and frequency point. First, to provide a general overview, and evaluate the location of the effect in terms of latency and frequency, we created a grand average power difference by computing the mean “real” power difference across all subjects and channels in the ROI. The same procedure was applied to the “surrogates”: we randomly picked one surrogate for each subject and averaged the information across ROI channels and subjects, and repeated this procedure 100,000 times to create a distribution of “surrogate grand-averages”. The strength of the “real” power difference was statistically assessed by applying a nonparametric randomization method allowing the identification of clusters on time-frequency points to control for multiple comparisons (Maris and Oostenveld, 2007). For both the “real” and “surrogate” averages, the time-frequency clusters were extracted by applying a two-sided 2.5% (arbitrary) threshold based on the distribution of “surrogate” dB values. Finally, we extracted the *p*-values for each “real” cluster as the proportion of “surrogates” cluster sums in the distribution above the cluster sum value of the real condition.

### Power dependent performance

In order to quantify the effect of alpha power on perception, we also tested how much variability in performance could be explained at the peak power difference. To this end, for each subject and channel, we sorted the trials based on the instantaneous reconstructed EEG power and split them into 5 (equally spaced) bins. We then extracted the normalized hit rate for each bin (correcting by the overall mean performance of the subject). A linear fit was applied to the mean across subjects for each channel, and the corrected slope was used as the percentage of performance modulation. We also report the R-square value for the goodness of fit.

## Results

To disentangle the relationship between alpha power and visual perception, we used white-noise sequences to constrain the state of background oscillatory activity. Instead of recording the EEG, we reconstructed the background oscillations by doing a convolution between the white-noise sequences and the impulse response functions recorded in a separate session (see section Method). This allowed us to evaluate how alpha power might be related to visual perception, independently of any impact of endogenous factors (not present in the IRF). Once the EEG had been reconstructed, we evaluated how the power of this signal might be related to the detection of near-perceptual threshold targets embedded in the WN sequences.

The target visibility was adjusted using a staircase procedure over the first 100 targets (~30 trials) to reach a 50% average detection rate, the achieved luminance contrast was then kept for the remainder of the experiment. During the remainder of the session, the hit rate stayed relatively stable, with subjects reaching an overall mean performance of 45.76% (standard deviation: 10.88%).

We then evaluated whether missed and seen target trials had different mean power across trials. Note that we limited our analysis to occipital and parietal channels, an a priori region of interest (see section Method). For each of the 22 channels in this ROI, we computed the power difference (in decibel) between the seen and unseen targets for each subject, channel and time-frequency point. First, we averaged the power difference across subjects and channels to get an overall idea of power influences on target detection. Using a permutation test and cluster correction, we found a significant difference between the mean power of trials where the target was missed vs. when the target was detected (significant after cluster correction, *p* < 0.000125) in the alpha band (from 3.46 to 19.28 Hz), just around the target presentation (from about −168.8 to 200 ms). The largest effect was at 11.68 Hz and −62.5 ms just before stimulus onset (see black dot on Figure 3).

Next we sought to quantify the variability in the behavioral responses that could be explained by power differences. Thus, we computed the normalized power dependent performance (i.e., percent hit rate corrected by the average hit rate across all bins) for each subject and channel in the ROI at the time of maximal power difference (11.68 Hz and −62.5 ms).

**Figure 4:**
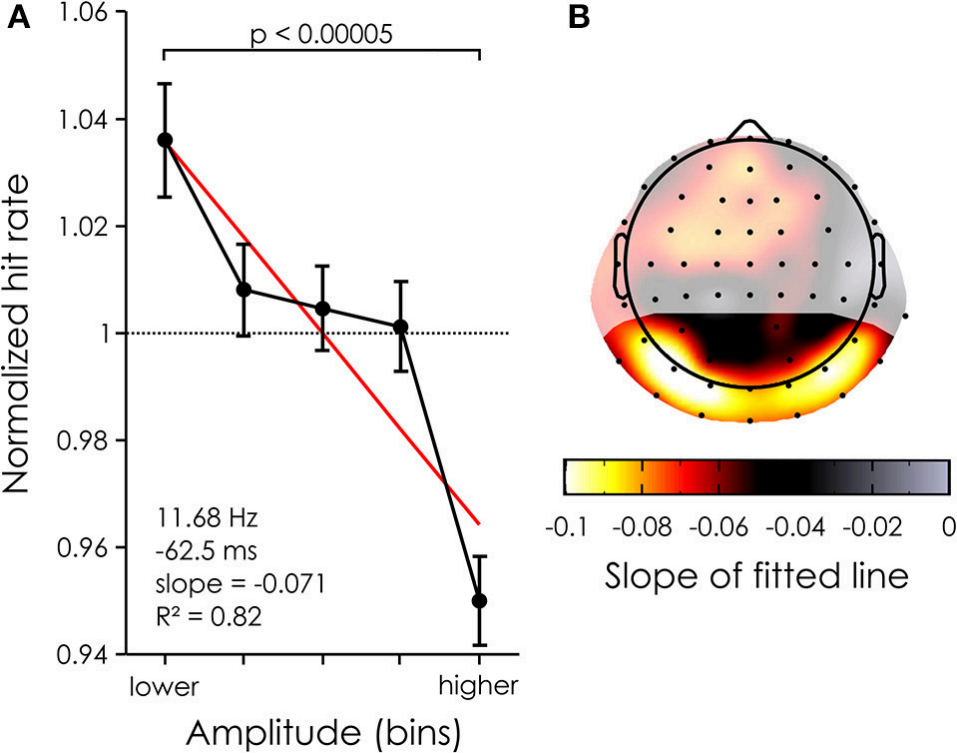
Power dependent performance. **(A)** Mean percent change averaged across all 22 channels in the ROI (black) and 20 subjects for each of 5 power bins. The bars represent the standard error of the mean. The red line represents the linear fit. **(B)** The mean power dependent performance was computed for each channel (averaged across subjects) and the coefficient of the linear fit was taken to represent the modulation of performance. The largest effects are found over the occipital channels. Shaded areas represent channels outside of the ROI.

There was a clear negative relationship between reconstructed EEG alpha power and target detection. A paired *t*-test revealed a significant difference in percent change [*p* < 0.00005, *t*_(19)_ = 5.1368, mean difference = 0.0860, 95% confidence interval of the difference = 0.0509–0.1210] between the bins with the highest and lowest power across all channels in the ROI (see Figure 4A), explaining ~7% of the variability in the behavior (slope of the linear fit = 0.071485). Note that the data was well approximated by a linear trend, as the fit had an r-squared of 0.82. At the single channel level, this effect was maximal over bilateral occipital channels, e.g., on channel PO7 where up to 10% of the variability in performance could be explained by alpha power differences (see Figure 4B). This normalized effect size of 10% implies that, for a subject whose average hit rate is about 50%, a 5% drop of hit rate will be seen from the lowest alpha bin to the highest (i.e., from 52.5 to 47.5% hit rate).

## Discussion

In this study, we examined the relationship between the power of ongoing oscillations and visual perception to better understand the correlates of sensory neural processing. We wished to disentangle the contribution of the two (direct/indirect) causal routes linking alpha oscillations and visual detection (Figure 1). Using the white-noise paradigm, we introduced modulations of alpha power independently of any fluctuations in endogenous factors. By using a linear model of brain activity, we were able to reconstruct (rather than record) the EEG around targets embedded within the WN sequences (Figure 2). Thus we had access to changes in the instantaneous state of the background oscillatory activity independently from confounding influence from endogenous factors (such as attention).

We found that some of the trial-by-trial variability in perception could be explained by fluctuations in the power of the reconstructed alpha oscillations at approximately −62 ms before target onset (Figures 3, 4). These effects are causal in nature: any modulation of alpha power present in the reconstructed EEG is, by design, constrained by the WN sequences. Consequently, any relationship with performance is necessarily a result of the WN sequences entraining the background oscillations in a predictable way. This is not to say that endogenous factors did not play a role in this experiment: it is still likely that fluctuations in endogenous factors had an impact on the behavioral response of subjects, through changes in attentiveness or arousal level, as the experiment unfolded. This idea is supported by the observation that alpha power-related performance modulations caused by attentional manipulations tend to be somewhat larger than the 5–10% change reported here. However, these fluctuations would be visible only in the recorded EEG (which we did not record in the second, critical session). The reconstructed EEG, on the other hand, only captures oscillations that are directly phase-locked to the background fluctuations in luminance values. It is thus virtually blind to these endogenous modulations. As such, our results contribute to a growing literature of studies that used rhythmic stimulation (either visual flicker, or transcranial rhythmic stimulation) to demonstrate that pre-stimulus alpha oscillations have a causal influence on visual perception, outside of any influence of endogenous factors (Ergenoglu et al., 2004; Hanslmayr et al., 2005, 2007; Romei et al., 2008a,b; van Dijk et al., 2008).

While endogenous factors could not explain the modulations of “reconstructed” alpha activity in our experiment (as explained above), there might still be room for attentional mechanisms to play a role in this task. Indeed, it is possible that the fluctuations in alpha power could themselves causally modulate attention. When the WN sequences induce higher alpha power, attention would become less efficient (and vice-versa for lower alpha power leading to improved attentional efficiency). These attentional fluctuations could in turn causally influence the perception of the targets. In other words, we cannot decide whether WN-induced fluctuations in alpha power *directly* influence perception, or whether they do so *indirectly* by modulating attention. In any case, this still allows us to rule out the “causal primacy” of attention and other endogenous factors.

More generally, our results are in line with the hypothesis that alpha oscillations play an active inhibitory role in shaping sensory processing (Klimesch et al., 2007; Jensen and Mazaheri, 2010; Foxe and Snyder, 2011), realized through a modulation of neuronal excitability (Haegens et al., 2015). In line with this idea, it has been suggested that the spontaneous fluctuations in alpha power presented in the Introduction could in fact reflect a spatial scanning mechanism, which would allow the detection of unpredictable events by randomly biasing neuronal activity at various locations in the visual field (Romei et al., 2008a). In fact, this is compatible with the spatio-temporal unfolding of the impulse response functions: IRFs recorded in response to lateralized stimuli show a systematic phase difference and “wave-like” propagation between contra- and ipsi-lateral cortex (Lozano-Soldevilla and VanRullen, 2017). This suggests that the impulse response function could highlight a scanning mechanism that is always present in the visual cortex (even in the absence of white-noise sequences), and implemented via a traveling alpha wave, as proposed 70 years ago by Pitts and McCulloch (1947).

In conclusion, using the WN paradigm to specifically extract background oscillatory activity, we show that alpha oscillations have a causal influence on visual target detection, independently from the effects of endogenous factors (such as attention), thus confirming the inhibitory role of alpha oscillations in visual perception.

## Author contributions

RV designed the research, SB implemented the research and collected the data, RV and SB analyzed data and wrote the paper together.

### Conflict of interest statement

The authors declare that the research was conducted in the absence of any commercial or financial relationships that could be construed as a potential conflict of interest.
